# Supplementary material for: A Dynamic Compartmental Model to Explore the Optimal Strategy of Varicella Vaccination: An Epidemiological Study in Jiangsu Province, China
Source: Trop Med Infect Dis. 2022 Dec 27;8(1):17. doi: 10.3390/tropicalmed8010017 (PMC9861499; doi:10.3390/tropicalmed8010017)
Supplement: Supplementary file 1 [file tropicalmed-08-00017-s001.zip › tropicalmed-2060727-supplementary.pdf]

Supplementary File S1: Full model description

Ordinary differential equations (S1) - (S7) represent 0-1-years-old, 1-3-years-old, 3-5 years-old, 5-10 years-old, 10-15 years-old and over 20 years-old respectively. Synthesizing the sub-models (S1) - (S7), we can get the full model of varicella transmission.

$$\begin{cases} \frac{dS_0(1,t)}{dt} = r_0(t) - \eta_1(1)S_0(1,t) - \beta(1)S_0(1,t)\sum_{k'=1}^{7} I(k',t) - S_0(1,t) - dS_0(1,t), \\ \frac{dS_1(1,t)}{dt} = \eta_1(1)(1-\varepsilon_1)S_0(1,t) - \beta(1)S_1(1,t)\sum_{k'=1}^{7} I(k',t) - S_1(1,t) - dS_1(1,t), \\ \frac{dI(1,t)}{dt} = \beta(1)\left(S_0(1,t) + S_1(1,t)\right)\sum_{k'=1}^{7} I(k',t) - cI(k,t) - dI(1,t), \\ \frac{dR(1,t)}{dt} = \eta_1(1)\varepsilon_1 S_0(1,t) + cI(1,t) - R(1,t) - dR(1,t). \end{cases} \tag{S1}$$

$$\begin{cases} \frac{dS_0(2,t)}{dt} = S_0(1,t) - \eta_1(2)S_0(2,t) - \beta(2)S_0(2,t)\sum_{k'=1}^{7} I(k',t) - \frac{1}{2}S_0(2,t) - dS_0(2,t), \\ \frac{dS_1(2,t)}{dt} = S_1(1,t) + \eta_1(2)(1-\varepsilon_1)S_0(2,t) - \eta_2(2)S_1(2,t) - \beta(2)S_1(2,t)\sum_{k'=1}^{7} I(k',t) - \frac{1}{2}S_1(2,t) - dS_1(2,t), \\ \frac{dS_2(2,t)}{dt} = \eta_2(2)(1-\varepsilon_2)S_1(2,t) - \beta(2)S_2(2,t)\sum_{k'=1}^{7} I(k',t) - \frac{1}{2}S_2(2,t) - dS_2(2,t), \\ \frac{dI(2,t)}{dt} = \beta(2)\left(S_0(2,t) + S_1(2,t) + S_2(2,t)\right)\sum_{k'=1}^{7} I(k',t) - cI(2,t) - dI(2,t), \\ \frac{dR(2,t)}{dt} = R(1,t) + \eta_1(2)\varepsilon_1 S_0(2,t) + \eta_2(2)\varepsilon_2 S_1(2,t) + cI(2,t) - \frac{1}{2}R(2,t) - dR(2,t). \end{cases} \tag{S2}$$

$$\begin{cases} \frac{dS_0(3,t)}{dt} = \frac{1}{2}S_0(2,t) - \eta_1(3)S_0(3,t) - \beta(3)S_0(3,t)\sum_{k'=1}^{7} I(k',t) - \frac{1}{2}S_0(3,t) - dS_0(3,t), \\ \frac{dS_1(3,t)}{dt} = \frac{1}{2}S_1(2,t) + \eta_1(3)(1-\varepsilon_1)S_0(3,t) - \eta_2(3)S_1(3,t) - \beta(k)S_1(k,t)\sum_{k'=1}^{7} I(k',t) - \frac{1}{2}S_1(3,t) - dS_1(3,t), \\ \frac{dS_2(3,t)}{dt} = \frac{1}{2}S_2(2,t) + \eta_2(3)(1-\varepsilon_2)S_1(3,t) - \beta(3)S_2(3,t)\sum_{k'=1}^{7} I(k',t) - \frac{1}{2}S_2(3,t) - dS_2(3,t), \\ \frac{dI(3,t)}{dt} = \beta(3)\left(S_0(3,t) + S_1(3,t) + S_2(3,t)\right)\sum_{k'=1}^{7} I(k',t) - cI(3,t) - dI(3,t), \\ \frac{dR(3,t)}{dt} = \frac{1}{2}R(2,t) + \eta_1(3)\varepsilon_1 S_0(3,t) + \eta_2(3)\varepsilon_2 S_1(3,t) + cI(3,t) - \frac{1}{2}R(3,t) - dR(3,t). \end{cases} \tag{S3}$$

$$\begin{cases} \dfrac{dS_0(4,t)}{dt} = \dfrac{1}{2}S_0(3,t) - \eta_1(4)S_0(4,t) - \beta(4)S_0(4,t)\sum_{k'=1}^{7} I(k',t) - \dfrac{1}{5}S_0(4,t) - dS_0(4,t), \\ \dfrac{dS_1(4,t)}{dt} = \dfrac{1}{2}S_1(3,t) + \eta_1(4)(1-\varepsilon_1)S_0(4,t) - \eta_2(4)S_1(4,t) - \beta(4)S_1(4,t)\sum_{k'=1}^{7} I(k',t) - \dfrac{1}{5}S_1(4,t) - dS_1(4,t), \\ \dfrac{dS_2(4,t)}{dt} = \dfrac{1}{2}S_2(3,t) + \eta_2(4)(1-\varepsilon_2)S_1(4,t) - \beta(4)S_2(4,t)\sum_{k'=1}^{7} I(k',t) - \dfrac{1}{5}S_2(4,t) - dS_2(4,t), \\ \dfrac{dI(4,t)}{dt} = \beta(4)\left(S_0(4,t) + S_1(4,t) + S_2(4,t)\right)\sum_{k'=1}^{7} I(k',t) - cI(4,t) - dI(4,t), \\ \dfrac{dR(4,t)}{dt} = \dfrac{1}{2}R(3,t) + \eta_1(4)\varepsilon_1 S_0(4,t) + \eta_2(4)\varepsilon_2 S_1(4,t) + cI(4,t) - \dfrac{1}{5}R(4,t) - dR(4,t). \end{cases} \tag{S4}$$

$$\begin{cases} \dfrac{dS_0(5,t)}{dt} = \dfrac{1}{5}S_0(4,t) - \beta(5)S_0(5,t)\sum_{k'=1}^{7} I(k',t) - \dfrac{1}{5}S_0(5,t) - dS_0(5,t), \\ \dfrac{dS_1(5,t)}{dt} = \dfrac{1}{5}S_1(4,t) - \beta(5)S_1(5,t)\sum_{k'=1}^{7} I(k',t) - \dfrac{1}{5}S_1(5,t) - dS_1(5,t), \\ \dfrac{dS_2(5,t)}{dt} = \dfrac{1}{5}S_2(4,t) - \beta(5)S_2(5,t)\sum_{k'=1}^{7} I(k',t) - \dfrac{1}{5}S_2(5,t) - dS_2(5,t), \\ \dfrac{dI(5,t)}{dt} = \beta(5)\left(S_0(5,t) + S_1(5,t) + S_2(5,t)\right)\sum_{k'=1}^{7} I(k',t) - cI(5,t) - dI(5,t), \\ \dfrac{dR(5,t)}{dt} = \dfrac{1}{5}R(4,t) + V_0^s(5,t) + V_1^s(5,t) + cI(5,t) - \dfrac{1}{5}R(5,t) - dR(5,t). \end{cases} \tag{S5}$$

$$\begin{cases} \dfrac{dS_0(6,t)}{dt} = \dfrac{1}{5}S_0(5,t) - \beta(6)S_0(6,t)\sum_{k'=1}^{7} I(k',t) - \dfrac{1}{5}S_0(6,t) - dS_0(6,t), \\ \dfrac{dS_1(6,t)}{dt} = \dfrac{1}{5}S_1(5,t) - \beta(6)S_1(6,t)\sum_{k'=1}^{7} I(k',t) - \dfrac{1}{5}S_1(6,t) - dS_1(6,t), \\ \dfrac{dS_2(6,t)}{dt} = \dfrac{1}{5}S_2(5,t) - \beta(6)S_2(6,t)\sum_{k'=1}^{7} I(k',t) - \dfrac{1}{5}S_2(6,t) - dS_2(6,t), \\ \dfrac{dI(6,t)}{dt} = \beta(6)\left(S_0(6,t) + S_1(6,t) + S_2(6,t)\right)\sum_{k'=1}^{7} I(k',t) - cI(6,t) - dI(6,t), \\ \dfrac{dR(6,t)}{dt} = \dfrac{1}{5}R(5,t) + cI(6,t) - \dfrac{1}{5}R(6,t) - dR(6,t). \end{cases} \tag{S6}$$

$$\begin{cases} \dfrac{dS_0(7,t)}{dt} = \dfrac{1}{5}S_0(6,t) - \beta(7)S_0(7,t)\sum_{k'=1}^{7} I(k',t) - dS_0(7,t), \\ \dfrac{dS_1(7,t)}{dt} = \dfrac{1}{5}S_1(6,t) - \beta(7)S_1(7,t)\sum_{k'=1}^{7} I(k',t) - dS_1(7,t), \\ \dfrac{dS_2(7,t)}{dt} = \dfrac{1}{5}S_2(6,t) - \beta(7)S_2(7,t)\sum_{k'=1}^{7} I(k',t) - dS_2(7,t), \\ \dfrac{dI(7,t)}{dt} = \beta(7)\left(S_0(7,t) + S_1(7,t) + S_2(7,t)\right)\sum_{k'=1}^{7} I(k',t) - cI(7,t) - dI(7,t), \\ \dfrac{dR(7,t)}{dt} = \dfrac{1}{5}R(6,t) + cI(7,t) - dR(7,t). \end{cases} \tag{S7}$$

Supplementary File S2: Parameters and initial conditions

Table S1. Parameters and initial conditions of the full model.

Model parameters	Source or range	Value
Demographic parameters		
Birth rate $r_0(t)$ (births/year):		
2006	[17]	899124
2007		904700
2008		918720
2009		933622
2010		965667
2011		800373
2012		694415
2013		694754
2014		716734
2015		717893
2016		715075
2017		699247
2018		720903
Mortality by age group d (1/year)	[17]	7×10^{-3}
Biological parameters		
Transfer rate from infected to recovered (c)	Calculated	11/12
Contact rate $\beta(k)$		
0-1	to be estimated	unknown
1-3		unknown
3-5		unknown
5-10		unknown
10-15		unknown
15-20		unknown
20+		unknown
Vaccine efficacy parameters		
Vaccine coverage of first dose η_1		
0-1 $\eta_1(0)$	to be estimated	unknown
1-3 $\eta_1(1)$		unknown
3-5 $\eta_1(2)$		unknown
5-10 $\eta_1(3)$		unknown
Vaccine coverage of second dose η_2		
1-3 $\eta_1(1)$	to be estimated	unknown
3-5 $\eta_1(2)$		unknown
5-10 $\eta_1(3)$		unknown
Vaccination effectiveness		
First vaccination ε_1	Assumption	60%
Second vaccination ε_2	Assumption	75%

Parameters of initial conditions		
The proportion of susceptible at each age-class [a]		
$\gamma(1)$		unknown
$\gamma(2)$		unknown
$\gamma(3)$		unknown
$\gamma(4)$	to be estimated	unknown
$\gamma(5)$		unknown
$\gamma(6)$		unknown
$\gamma(7)$		unknown
The proportion of subgroups in susceptible [b, c]		
$\alpha_0(1)$		unknown
$\alpha_0(2)$		unknown
$\alpha_1(2)$		unknown
$\alpha_0(3)$	to be estimated	unknown
$\alpha_1(3)$		unknown
$\alpha_0(4)$		unknown
$\alpha_1(4)$		unknown

[a] $\gamma(k)$ represents the proportion of susceptible in the total population in k^{th} age group, i.e., $S(k,0) = \gamma(k)N(k,0)$

[b] $\alpha_i(k)$ represents the proportion in k^{th} age group susceptible persons who received i^{th} vaccination but fail, $i = 0,1,2$.

[c] Because the second vaccination is not permitted for susceptible whose age is less than one year old, $\alpha_1(1)$ is taken as constant 1.

Table S2. Transfer parameters related to age growth in each age group [a].

Age group	$F_0(k,t)$	$F_1(k,t)$	$F_2(k,t)$	$F_R(k,t)$
Newborns	$r_0(t)$	0	0	0
0-1 year-old	$S_0(1,t)$	$S_1(1,t)$	0	$R(1,t)$
1-3 years-old	$\frac{1}{2}S_0(2,t)$	$\frac{1}{2}S_1(2,t)$	$\frac{1}{2}S_2(2,t)$	$\frac{1}{2}R(2,t)$
3-5 years-old	$\frac{1}{2}S_0(3,t)$	$\frac{1}{2}S_1(3,t)$	$\frac{1}{2}S_2(3,t)$	$\frac{1}{2}R(3,t)$
5-10 years-old	$\frac{1}{5}S_0(4,t)$	$\frac{1}{5}S_1(4,t)$	$\frac{1}{5}S_2(4,t)$	$\frac{1}{5}R(4,t)$
10-15 years-old	$\frac{1}{5}S_0(5,t)$	$\frac{1}{5}S_1(5,t)$	$\frac{1}{5}S_2(5,t)$	$\frac{1}{5}R(5,t)$
15-20 years-old	$\frac{1}{5}S_0(6,t)$	$\frac{1}{5}S_1(6,t)$	$\frac{1}{5}S_2(6,t)$	$\frac{1}{5}R(6,t)$
Over 20 years [b] old	0	0	0	0

[a] we simply take the reciprocal of interval length of each age group as the transfer rate of each age group.

[b] There is no age-dependent transfer in the age group over 20 years old.

Supplementary File S3: Parameter estimation

The variance of measured components, $I(k,t), k=1,2,\cdots,7$, was given by an inverse gamma distribution with hyperparameters (0.01, 4), where 0.01 is the initial error variance which is updated by the inverse gamma distribution[30] and the small MCMC package provided in this website was used to estimate the parameters. In estimating the unknown parameters and initial conditions of the full model, it is recommended to select multivariate normal distribution as priori distribution with limited range information in Table S1, which can ensure the good convergence of MCMC analysis.

After fitting the model with the survey data of varicella cases in Jiangsu Province from 2005 to 2018, the estimated medians with their corresponding first and third quartiles (Q1-Q3) of the undetermined parameters are listed in Table S3. Figure S1 is the distribution diagram of each undetermined parameter.

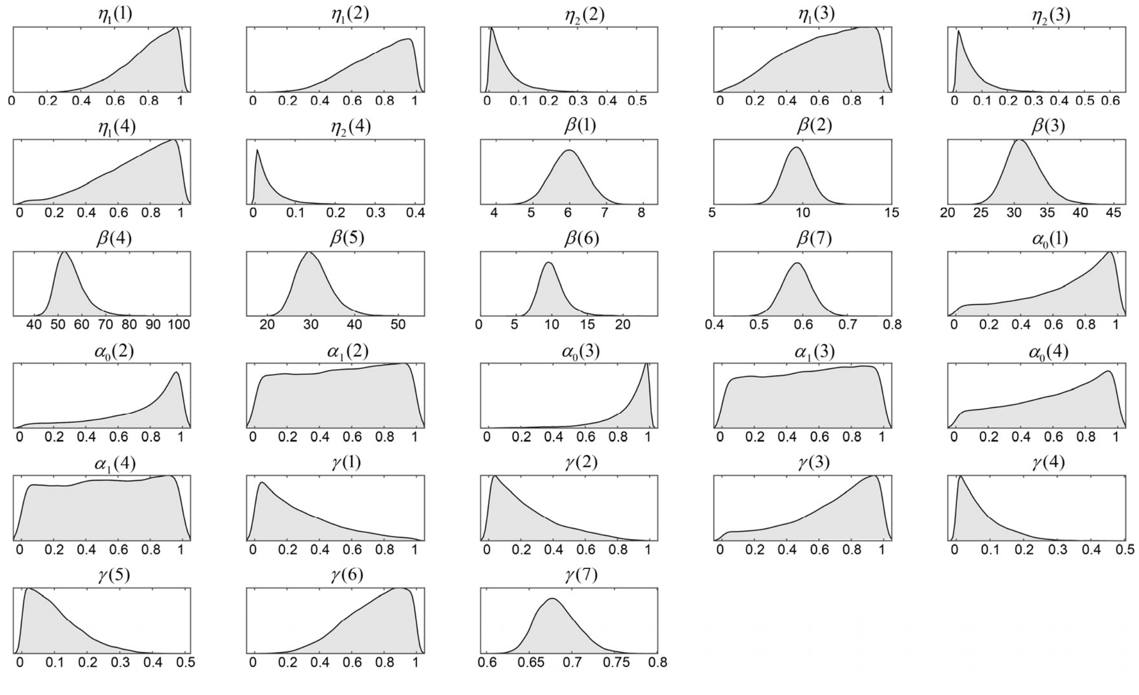

Figure S1. Distribution of each undetermined parameter in full model based on MCMC analysis. The algorithm runs for 2×10^6 iterations with a burn-in of 10^6 iterations, and the Geweke convergence diagnostic method was employed to assess convergence of chains. The initial values of each parameter were randomly selected within their feasible ranges in Table S1.

Table S3. Estimated parameters in model (1.1).

Parameter	Median (Q1, Q3)	Parameter	Median (Q1, Q3)
$\eta_1(1)$	0.830(0.710,0.921)	$\alpha_1(1)$	0.745(0.492,0.898)
$\eta_1(2)$	0.785(0.635,0.899)	$\alpha_1(2)$	0.828(0.603,0.937)
$\eta_2(2)$	0.037(0.015,0.076)	$\alpha_2(2)$	0.533(0.271,0.774)
$\eta_1(3)$	0.664(0.462,0.838)	$\alpha_1(3)$	0.916(0.817,0.967)
$\eta_2(3)$	0.041(0.016,0.083)	$\alpha_2(3)$	0.535(0.275,0.773)
$\eta_1(4)$	0.744(0.557,0.882)	$\alpha_1(4)$	0.664(0.397,0.858)
$\eta_2(4)$	0.023(0.009,0.048)	$\alpha_2(4)$	0.521(0.266,0.769)
$\beta(1)\times10^{-8}$	5.970(5.648,6.289)	$\gamma(1)$	0.215(0.090,0.405)
$\beta(2)\times10^{-8}$	9.646(9.148,10.157)	$\gamma(2)$	0.184(0.079,0.347)
$\beta(3)\times10^{-8}$	31.276(29.647,33.133)	$\gamma(3)$	0.749(0.541,0.886)
$\beta(4)\times10^{-8}$	54.268(51.007,58.374)	$\gamma(4)$	0.054(0.023,0.104)
$\beta(5)\times10^{-8}$	30.253(27.876,32.951)	$\gamma(5)$	0.085(0.040,0.146)
$\beta(6)\times10^{-8}$	9.852(8.825,11.049)	$\gamma(6)$	0.767(0.617,0.885)
$\beta(7)\times10^{-8}$	0.586(0.565,0.608)	$\gamma(7)$	0.681(0.665,0.698)

Supplementary File S4: Basic reproduction number

According to [20], the basic reproduction number of the full model can be defined by the spectral radius of the next infection operator. The main steps are described as follows:

Firstly, the expression of disease-free equilibrium $E_0=\left(S_0^*(k),S_1^*(k),S_2^*(k),I^*(k),R^*(k)\right)$, $k=1,2,3,\cdots,7$ of the full model is obtained, in which

$$S_0^*(1)=\frac{r_0}{1+d+\eta_1(1)},\quad S_1^*(1)=\frac{S_0^*(1)\eta_1(1)(1-\varepsilon_1)}{1+d},\quad S_2^*(1)=0,$$

$$S_0^*(2)=\frac{S_0^*(1)}{\frac{1}{2}+d+\eta_1(2)},\quad S_1^*(2)=\frac{S_0^*(2)\eta_1(2)(1-\varepsilon_1)+S_1^*(1)}{\frac{1}{2}+d+\eta_2(2)},\quad S_2^*(2)=\frac{S_1^*(2)\eta_2(2)(1-\varepsilon_2)}{\frac{1}{2}+d},$$

$$S_0^*(3)=\frac{\frac{1}{2}S_0^*(2)}{\frac{1}{2}+d+\eta_1(3)},\quad S_1^*(3)=\frac{S_0^*(3)\eta_1(3)(1-\varepsilon_1)+\frac{1}{2}S_1^*(2)}{\frac{1}{2}+d+\eta_2(3)},\quad S_2^*(3)=\frac{S_1^*(3)\eta_2(3)(1-\varepsilon_2)+\frac{1}{2}S_2^*(2)}{\frac{1}{2}+d},$$

$$S_0^*(4)=\frac{\frac{1}{2}S_0^*(3)}{\frac{1}{5}+d+\eta_1(4)},\quad S_1^*(4)=\frac{S_0^*(4)\eta_1(4)(1-\varepsilon_1)+\frac{1}{2}S_1^*(3)}{\frac{1}{5}+d+\eta_2(4)},\quad S_2^*(4)=\frac{S_1^*(4)\eta_2(4)(1-\varepsilon_2)+\frac{1}{2}S_2^*(3)}{\frac{1}{5}+d},$$

$$S_0^*(5)=\frac{\frac{1}{5}S_0^*(4)}{\frac{1}{5}+d},\quad S_1^*(5)=\frac{\frac{1}{5}S_1^*(4)}{\frac{1}{5}+d},\quad S_2^*(5)=\frac{\frac{1}{5}S_2^*(4)}{\frac{1}{5}+d},$$

$$S_0^*(6)=\frac{\frac{1}{5}S_0^*(5)}{\frac{1}{5}+d},\quad S_1^*(6)=\frac{\frac{1}{5}S_1^*(5)}{\frac{1}{5}+d},\quad S_2^*(6)=\frac{\frac{1}{5}S_2^*(5)}{\frac{1}{5}+d},$$

$$S_0^*(7)=\frac{\frac{1}{5}S_0^*(6)}{d},\quad S_1^*(7)=\frac{\frac{1}{5}S_1^*(6)}{d},\quad S_2^*(7)=\frac{\frac{1}{5}S_2^*(6)}{d},$$

$$I^*(1)=0,\quad I^*(2)=0,\quad I^*(3)=0,\quad I^*(4)=0,\quad I^*(5)=0,\quad I^*(6)=0,\quad I^*(7)=0,$$

$$R^*(1)=\frac{S_0^*(1)\eta_1(1)\varepsilon_1}{1+d},\quad R^*(2)=\frac{R^*(1)+S_0^*(2)\eta_1(2)\varepsilon_1+S_1^*(2)\eta_2(2)\varepsilon_2}{\frac{1}{2}+d}$$

$$R^*(3)=\frac{\frac{1}{2}R^*(2)+S_0^*(3)\eta_1(3)\varepsilon_1+S_1^*(3)\eta_2(3)\varepsilon_2}{\frac{1}{2}+d},\quad R^*(4)=\frac{\frac{1}{2}R^*(3)+S_0^*(4)\eta_1(4)\varepsilon_1+S_1^*(4)\eta_2(4)\varepsilon_2}{\frac{1}{5}+d},$$

$$R^*(5)=\frac{\frac{1}{5}R^*(4)}{\frac{1}{5}+d},\quad R^*(6)=\frac{\frac{1}{5}R^*(5)}{\frac{1}{5}+d},\quad R^*(7)=\frac{\frac{1}{5}R^*(6)}{d}.$$

Secondly, according to the calculation procedure of basic reproduction number for multi-group model, two matrix were obtained,

$$F=\left[\beta(k)\left(S_0^*(k)+S_1^*(k)+S_2^*(k)\right)\right],\quad V=\left[(c+d)\delta_{kk'}\right],$$

here $\delta_{kk'}=\begin{cases}1, k=k'\\ 0, k\neq k'\end{cases}, k=1,2,\cdots,7$.

Thirdly, following Diekmann *et al* [31], we call

$$FV^{-1}=\left[\frac{\beta(k)\left(S_0^*(k)+S_1^*(k)+S_2^*(k)\right)\delta_{kk'}}{c+d}\right], k=1,2,\cdots,7$$

the next generation matrix of the full model.

Lastly, the basic reproduction number is acquired by

$$R_0=\rho\left(FV^{-1}\right)=\sum_{k=1}^{7}R_0(k)=\sum_{k=1}^{7}\frac{\beta(k)\left(S_0^*(k)+S_1^*(k)+S_2^*(k)\right)}{c+d}.$$

Supplementary File S5: Global sensitivity analysis

Because the basic reproduction number represents the epidemic intensity of the disease, we took it as a dependent variable in Latin Hypercube Sampling (LHS). Partial rank correlation coefficients (PRCCs) was used to examine the global sensitivity of the parameters in the model.

The distribution for parameters of vaccination coverage rate, contact rate (with the median values) and corresponding first and third quartiles (Q1-Q3) were given in Table S3 and Figure S1. Because no prior distribution was obtained for the parameters of vaccination efficacy, we set the uniform distribution within ±20% of the corresponding median [21]. According to previous study [22], we considered an absolute values of PRCC > 0.4 indicative of strong correlation between the input parameters and the output variables, values between 0.2 and 0.4 as moderate or weak correlations, and values between 0 and 0.2 as not significantly different from zero.

Clearly, expanding vaccination coverage, reducing the contact frequency with infected population, and improving vaccination efficacy are all potentially feasible strategies for better preventing and controlling varicella epidemics. We further explored the influence of these three kinds of parameters on the size of basic reproduction number. Because epidemiological survey estimated that the first vaccination coverage rate was significantly higher than the second, which is also confirmed by the model fitting parameters (about 65% vs 5%), we only investigated the effect of the second vaccination coverage rate on the basic reproduction number. Related parameters ($\eta_2(2)$, $\eta_2(3)$, $\eta_2(4)$) were set to vary synchronously within the range of 1 to 10 times of their medians. Parameters of vaccination efficacy and efficacy contact rate varied synchronously within the range of 50% to 100% and 100% to 150% of their medians, respectively. During the simulation, once a parameter reached its upper/lower limit value (Table S1), the upper/lower limit value was given to the parameter.
